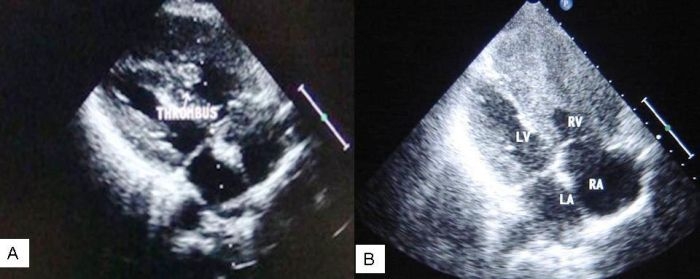# Right Ventricular Endomyocardial Fibrosis Presenting With Ventricular Tachycardia And Apical Thrombus - An Interesting Presentation

**Published:** 2009-11-01

**Authors:** Amitesh Aggarwal, Bineet Sinha, Surender Rajpal, Shridhar Dwivedi, Vishal Sharma

**Affiliations:** Department of Medicine/ Preventive Cardiology, University College of Medical Sciences, University of Delhi & GTB Hospital, Delhi-110095.

**Keywords:** Endomyocardial Fibrosis, Ventricular Tachycardia

## Abstract

Endomyocardial fibrosis is a progressive disease of unknown origin affecting children and young adults. It involves inflow portion of right and/or left ventricle and apex. It may be associated with thrombus. Literature regarding right ventricular endomyocardial fibrosis with thrombus is scarce. Here we report a rare case of right ventricular endomyocardial fibrosis presenting as ventricular tachycardia and echocardiographic evidence of apical thrombus. Interestingly there was no pulmonary involvement or evidence of deep venous thrombosis. This case also underscores the importance of urgent echocardiography in diagnosis of obscure cases of ventricular tachycardia.

## Case Summary

A 36-yr-old female presented to emergency with the complaint of sudden onset loss of consciousness for 2-3 minutes. She spontaneously regained her consciousness after this episode. Her unconsciousness was not associated with trauma, seizures, focal neurological deficits, confusion, chest pain, palpitation, fever, diarrhea or vomiting. She had no past history of coronary artery disease, cerebrovascular accident, seizure disorder, tuberculosis, hypertension or similar episode of unconsciousness. She had two children, both delivered at full term. The last child birth was 7 years back. There was no history of abortions or stillbirth. She denied any intake of oral contraceptives, or any other medication She was non alcoholic and non smoker. There was no history of sudden cardiac death or similar disease in the family.  On presentation her pulse was rapid and feeble with monitor showing heart rate of 300/min, blood pressure of 60 mm of Hg systolic and respiratory rate of 30/min.

**Figure 1:**
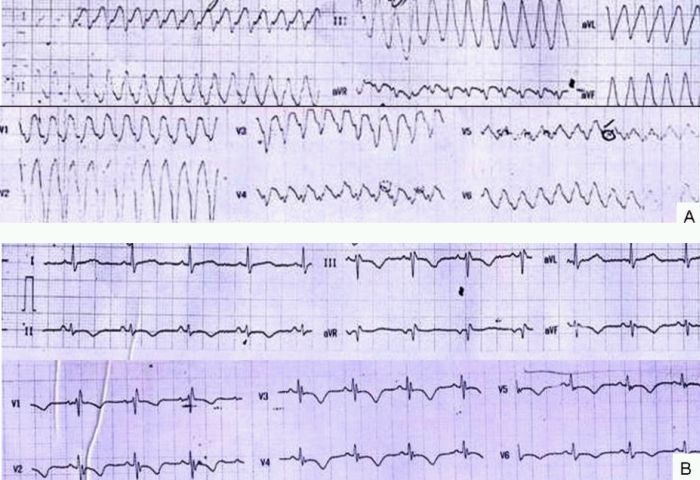


ECG showed evidence of ventricular tachycardia (VT) with an inferior axis and left bundle branch morphology (Figure 1A) for which she was given 360 J of synchronized DC shock with subsequent reversion to normal sinus rhythm and stabilization of blood pressure. The QTc was 0.447 secRepeat ECG showed right bundle branch block with T wave inversion in leads V2-V6, II, III, aVF with left axis deviation (Figure 1B). An urgent echocardiography was performed which showed obliteration of the right ventricular apex, a thrombus lodged at the apical area, restrictive flow pattern across the tricuspid valve, enhanced density of the right intraventricular band and enlarged right atrium that was suggestive of right ventricular endomyocardial fibrosis with apical thrombus with normal pulmonary valves and no regional wall motion abnormality (Figure 2A). The Doppler TR gradient was 22 mm of Hg. Her baseline serum biochemistry including thyroid profile, lipid profile and blood sugar was normal. CPK-MB was not raised and Troponin T was negative. Her hs-CRP (24.30mg/L), homocysteine (16.46µmol/L), fibrinogen (571mg/dl) and D dimer (18.79µg/ml) were elevated. Her antiphospholipid IgG and IgM antibodies; Protein C, functional and Protein S, functional were within normal range. Contrast enhanced computed tomography of chest showed no evidence of pulmonary thromboembolism, pulmonary hypertension or parenchymal involvement. Doppler ultrasound of bilateral lower limbs and carotid was normal. Based on the above clinical features and criteria for diagnosis and assessment of severity of endomyocardial fibrosis [1] (i.e 2 major or 1 major and 2 minor criteria) a diagnosis of moderate right ventricular endomyocardial fibrosis was established. These criteria have been validated in a study on 1063 subjects from rural Mozambique [1]. She was treated with anticoagulants (warfarin) and amiodarone. She improved on this treatment. Subsequent echocardiography revealed decrease in size of right ventricular thrombus (Figure 2B). Patient is still on regular follow up.

## Discussion

Endomyocardial fibrosis is a relatively frequent cause of heart failure and death in African countries [2]. However, its occurrence in most parts of India and Asian countries is uncommon [3]. It is characterized by fibrous endocardial lesions of the inflow of the right or left ventricle or both and often involves in atrioventricular valves resulting in regurgitation [4]. The disease affects both sexes equally and is common in children and young adults. Combined right and left ventricular disease occurs in about 50% cases with pure left ventricular involvement occurring in 40% and pure right ventricular involvement in the remaining 10% population [5]. Pure right ventricular involvement usually shows extensive fibrous thickening of the inflow tract and apex with a mass of thrombus [4]. The echocardiographic criteria for diagnosis include 6 major criteria (endomyocardial plaques >2 mm, thin (=1 mm) endomyocardial patches in  more than one area of ventricular wall, obliteration of ventricular apex, thrombi without severe ventricular dysfunction, right ventricular apical notch and AV valve dysfunction secondary to valvular adhesion to ventricular wall) and 7 minor criteria (one ventricular wall affected by endomyocardial patches,  restrictive flow across atriventricular valves, diastolic opening of pulmonary valve, diffuse thickening of the anterior mitral leaflet, enlarged atrium with normal-size ventricle, M-movement of the interventricular septum and flat posterior wall, and increased density of the moderator or other intraventricular bands). To establish the diagnosis of endomyocardial fibrosis 2 major or 1 major and 2 minor criteria are needed [4]. Our patient clearly had 2 major criteria including obliteration of right ventricular apex and right ventricular thrombus. Three minor criteria including restrictive flow pattern across the tricuspid valve, enhanced density of the right intraventricular band and enlarged right atrium were also seen. The severity score was 13 indicative of moderate disease.

Besides endomyocardial fibrosis right ventricular thrombus has also been seen in a variety of other clinical conditions such as hypercoagulable state, i.e. protein C and S deficiency [6], Behcet's disease [7], Loffler endocarditis [8], amyloidotic heart disease  [9] etc. However in our case the above clinical conditions were ruled out by appropriate investigations as cited above.

The mainstay of treatment of endomyocardial fibrosis is surgical excision of fibrous endocardium and replacement of damaged valves [10]. When surgery is contraindicated or denied by the patient, medical therapy should be opted. If right or left ventricular failure sets in, digitalis and diuretics should be added  [11,12]. In case of intramural thrombus, anticoagulation should be initiated if no contraindication exists. Our patient presented for the first time with ventricular tachycardia which was later found to be associated with right ventricular endomyocardial fibrosis and apical thrombus. She was started on anticoagulants and anti-arrhythmics after which she became asymptomatic. The present case illustrates the importance of urgent echo in diagnosing obscure causes of ventricular tachycardia presenting in emergency.

## Figures and Tables

**Figure 2:**